# Neovascular Age-Related Macular Degeneration in the Very Old (≥90 Years): Epidemiology, Adherence to Treatment, and Comparison of Efficacy

**DOI:** 10.1155/2017/7194927

**Published:** 2017-06-04

**Authors:** Yousif Subhi, Torben Lykke Sørensen

**Affiliations:** ^1^Clinical Eye Research Division, Department of Ophthalmology, Zealand University Hospital, Roskilde, Denmark; ^2^Faculty of Health and Medical Sciences, University of Copenhagen, Copenhagen, Denmark

## Abstract

**Purpose:**

To investigate neovascular age-related macular degeneration (AMD) in patients aged ≥90 years from several perspectives for a comprehensive overview: prevalence, presenting characteristics, treatment adherence, reasons for discontinuation, and efficacy of antivascular endothelial growth factor (VEGF) treatment comparing Ranibizumab and Aflibercept.

**Methods:**

In this retrospective chart review, we determined the prevalence and presenting characteristics by reviewing all data for patients referred to our department with treatment-naïve neovascular AMD. By looking at historical cohorts, we determined adherence to treatment, reasons for discontinuation, and treatment outcomes after loading dose, 12 months, and 24 months.

**Results:**

Patients aged ≥90 years constituted 7% of the patients. Treatment was discontinued in 51%, primarily because of death and treatment burden. Mean change in best-corrected visual acuity was 3.2, 1.5, and −2.2 ETDRS letters at 4, 12, and 24 months, respectively. Aflibercept was superior to Ranibizumab in visual and anatomic outcomes. After two years of treatment, patients losing ≥15 ETDRS letters made up 19% in the Aflibercept group and 26% in the Ranibizumab group.

**Conclusions:**

We propose that the very old patients with neovascular AMD may constitute a distinctive group warranting special attention and possibilities for individualized therapy. Possible differences between anti-VEGF agents need further investigations.

## 1. Introduction

Age-related macular degeneration (AMD) is the most common reason for irreversible vision loss in the developed world [1]. Neovascular AMD represents a late stage of the disease, where vascular endothelial growth factor (VEGF) mediated development of choroidal neovascularizations (CNV) [2]. From only being able to postpone the inevitable scarring of the macula and loss of central vision, we can now improve or at least stabilize visual acuity and visual function in the majority of patients by the widespread introduction of anti-VEGF treatment [3–5].

The pivotal phase 3 trials MARINA and ANCHOR studies demonstrated that monthly Ranizbiumab (Lucentis, Novartis International AG, Basel, Switzerland) improved mean visual acuity for all subtypes of neovascular AMD [6, 7]. Its efficacy was later compared to that of Aflibercept (Eylea, Bayer AG, Leverkusen, Germany) in the noninferiority VIEW trails, where Aflibercept treatment was able to reach similar outcomes [8]. Aflibercept is similar to Ranibizumab in its pharmacodynamic properties but differs in its pharmacokinetics due to a VEGF-trap design that enables treatment with potentially 8 weeks' intervals. Many clinical and observational studies have confirmed the efficacy of Ranibizumab and Aflibercept and explored the possibility individualizing therapy depending on the patient's characteristics [9, 10]. One interesting aspect is how anti-VEGF efficacy is affected by *age*. Several studies find that the change in best-corrected visual acuity (BCVA) is negatively correlated with age [11–22]. Subgroup analyses of the two years outcomes in the MARINA study found that unlike the younger patients which experienced a significant improvement of BCVA, patients ≥85 experienced no change in BCVA [13]. Similar results were reported for the first-year outcomes of the ANCHOR study [14]. Considering that anti-VEGF treatment experiences from daily clinical practice can be somewhat less promising than that seen in clinical trials [3], no change in BCVA may be hard to achieve in this patient group.

Ageing is an inevitable biological process that significantly affects the cells and organs of the body. Macula undergoes a range of changes [23]. The number of retinal pigment epithelium (RPE) cells decreases while the number of photoreceptors remains relatively stable [24]. Thus, each RPE cell must support more photoreceptors, which increases the metabolic and phagocytic stress on the RPE cells by which they respond by increasing in size and becoming multinucleated [25]. These aged and stressed RPE cells develop a higher VEGF response [26]. Taken together, increasing age leads to increased fragility of the macula [23], which we speculate may be a part of the reason for why the oldest individuals experience worse treatment response.

The average life expectancy is 80 years in developed countries such as Denmark, but diving into the statistics reveals that although 60+ years old individuals constitute 25%—every fourth—of the population, only <1% reaches 90+ years of age [27]. These very old individuals (defined as having aged ≥90 years) represent a small group that may significantly differ from the rest in terms of macular biology. However, another important aspect is also their opinion on the need for treatment and the burden of treatment in a monthly/bimonthly treatment regime. In cancer research, it is a well-documented phenomenon that some elderly refuse treatment because the perceived gain life expectancy does not outweigh the loss in quality of life [28]. Therefore, there may be several aspects among the very old patients with neovascular AMD with potential to influence the treatment response.

In this study, we investigated neovascular AMD in patients aged ≥90 years from several perspectives to give a comprehensive overview. First, we looked at their overall prevalence in a large tertiary retinal center and their presenting characteristics. Then, we looked at their adherence to anti-VEGF treatment and reasons for discontinuation. Finally, we evaluated results from anti-VEGF treatment and compared efficacy of Ranibizumab and Aflibercept using historical cohorts where each was the primary choice of treatment.

## 2. Materials and Methods

### 2.1. Study Design and Patient Eligibility

This study is a retrospective review of patients attending the Department of Ophthalmology at Zealand University Hospital, Roskilde, Denmark. All aspects of this study were conducted in accordance with the ethical principles stated in the Declaration of Helsinki. According to the national law and local hospital research guidelines, no institutional review board approval is required for retrospective observational clinical studies that review routine clinical practice.

We first determined the epidemiological aspects of patients with neovascular AMD aged ≥90 years: the prevalence and their presenting characteristics. For this part of the study, we reviewed all data for patients referred to our department in the year 2014. Eligible for analyses were patients with treatment-naïve neovascular AMD regardless of the status of their lesion (i.e., whether or not it was deemed treatable) or enrollment to anti-VEGF treatment. Diseases that share features with neovascular AMD (e.g., polypoidal choroidal vasculopathy) were not included.

Then, we looked at adherence to treatment, reasons for discontinuation, and treatment outcomes for a 2-year period comparing Aflibercept and Ranibizumab. For this part of the study, we included treatment results from all our patients aged ≥90 years diagnosed with treatment-naïve neovascular AMD from 2009 to 2012 (where Ranibizumab was the primary choice of treatment) and from 2014 to 2015 (where Aflibercept was the primary choice of treatment). In 2013 where Aflibercept was introduced in our clinic, we were concerned about the ongoing discussions about Aflibercept increasing the risk of cerebrovascular events [29] and allocated patients with selected comorbidities to Ranibizumab treatment. Hence, we did not include patients from 2013 to avoid selection bias.

### 2.2. Access to Retinal Care

In Denmark, primary sector units (practicing ophthalmologists and general practitioners) and hospital departments refer all patients suspected of neovascular AMD to ophthalmology departments since this is the only access to free-of-charge anti-VEGF treatment. Within 1-2 workdays after receiving an electronic referral, the patient is invited and booked for detailed retinal diagnosis. From the time of booking, the patient is in most cases seen within two weeks, depending on the patient's availability and the availability of the department. Patients are offered free-of-charge transportation between their home and the hospital department, including patients that may need wheelchair or special support.

### 2.3. Retinal Diagnosis

All patients referred for retinal diagnosis undergo a comprehensive ophthalmic examination including dilated fundus examination, measurement of best-corrected visual acuity (BCVA) in each eye using the Early Treatment of Diabetic Retinopathy Study (ETDRS) chart [30], retinal imaging using Heidelberg HRA-Spectralis Spectral Domain optical coherence tomography (OCT) (Heidelberg Engineering, Heidelberg, Germany), and retinal angiography using fluorescein and indocyanine green. All BCVAs were measured by personnel specifically trained in ETDRS measurements. We used CC-100 charts (Topcon Corp., Tokyo, Japan). All patients started from the top of the chart and read each letter of each horizontal line and progressed downwards until reaching a row where a minimum of three letters on a line could not be read. Each eye was tested individually and was scored according to correctly identified number of letters. Patients read the chart at 4 meters (adding 45 letters to the final score), but if the patient was unable to read the letters, then the chart was read at 1 meter (adding 15 letters to the final score).

Treatment was initiated in eyes with neovascular AMD as defined in the Clinical Age-Related Maculopathy Staging System (grade 5) [31] and in the presence of active CNV and no predominance of fibrosis and/or atrophy. In other words, this included active subfoveal CNV as evaluated by the presence of leakage in fluorescence angiography with the presence of one or more characteristics such as hemorrhage, exudates, serous detachments, and intraretinal edema. If the BCVA was ≥20 ETDRS letters or the patient had cardiovascular events within ≤3 months, treatment was commenced on a case-by-case basis with special emphasis on the state of the patient's contralateral eye.

### 2.4. Treatment and Follow-Up

All eyes received three consecutive monthly intravitreal injections with either Aflibercept (0.05 mL) or Ranibizumab (0.05 mL). Choice of Aflibercept or Ranibizumab was based on our local guidelines which were Ranibizumab prior to widescale Aflibercept introduction in 2013 and Aflibercept as first line of treatment starting from 2014 based on national guidelines for the treatment of neovascular AMD. Injections were given by physicians or specially trained injection nurses [32]. After the third injection, the patients were reevaluated in follow-ups using dilated fundus examination, measurement of best-corrected visual acuity (BCVA), and OCT scans to determine whether or not the macula was dry so that additional injections may be warranted. The reevaluation was after 4 weeks for Ranibizumab and 8 weeks for Aflibercept. We followed a pro re nata (PRN) treatment regime for both Aflibercept and Ranibizumab. Retreatment criteria, based on local guidelines and recommendations from the Danish Ophthalmological Society, were the same during the study period (2009 to 2017) [33]: the presence of subretinal or intraretinal fluid on OCT or retinal hemorrhage either new or persisting. In case of loss of visual acuity with no subretinal or intraretinal fluid, we repeated retinal angiography with fluorescein and indocyanine green to evaluate the presence of active CNV, which was another retreatment criterion. In eyes with development of untreatable retinal tubuli or fibrotic scar, or BCVA < 20 ETDRS letters with a dry macula, treatment was stopped. If additional injections were needed, the patient was booked for two to three anti-VEGF injections and reevaluated. Injections after the loading dose phase were given with 4 weeks intervals for Ranibizumab and 8 weeks for Aflibercept. If no additional injections were needed due to a dry macula, we booked the patient for reevaluation. After consecutive reevaluations with a dry macula, the disease was considered provisionally inactive and the patient was referred to a local primary sector ophthalmologist for future controls.

### 2.5. Data Analysis and Statistics

The prevalence of patients with neovascular AMD with an age ≥ 90 was calculated including a confidence interval for the prevalence estimate with a continuity correction [34]. We reviewed the identified patients' clinical characteristics using OCTs, angiographies, and BCVA. BCVA was obtained from treatment databases.

Categorical variables are presented using numbers and percentages and compared using the *χ*^2^-test and Fisher's exact test in case any subcategory had *n* < 5. Continuous variables were checked for normal distribution using the Kolmogorov-Smirnov test. Where normal distribution was present, data was presented using mean and standard deviation (SD) and tested using parametric tests. Age did not fit normal distribution and was right tailed, so this parameter was presented in median and interquartile range (IQR) and tested using Mann–Whitney *U* test.

Using data from eligible patients enrolled between 2009–2012 and 2014-2015, we determined which patients discontinued treatment and noted the reason. Based on different reasons for discontinuation, we explored adherence to treatment over time using a time-to-event curve. We defined the start point as date of treatment start and followed the patient until either event or censoring. Time-to-event curves were made specifically for each reason for discontinuation by censoring the other reasons.

Visual and anatomical treatment outcomes were measured as change in BCVA and average central retinal thickness (CRT) after approximately 4 months (first reevaulation after treatment commencement), 12 months, and 24 months. The CRT was defined as the average thickness of an area with a diameter of 1 mm around the fovea including any subretinal hyperreflective material. Due to different reasons for treatment discontinuation, we analyzed data using the last observation carried forward (LOCF) method to account for missing data for all patients that had at least one reevaluation after treatment commencement. LOCF was not made for 24 months follow-up for patients started in treatment in 2015 since most of these patients did not have their follow-up at time of analysis (March 2017). We assumed that using the LOCF on these patients would bias the results towards better outcomes in the Aflibercept groups because 12 months outcomes in general are better than 24 months outcomes.

When a patient's both eyes were eligible for our analyses, we only included data from one eye (the first eye diagnosed with neovascular AMD or the right eye in case both eyes were diagnosed simultaneously) to avoid statistical problems with assumptions of independent sampling. In the analyses, we first looked at whether the change in BCVA and average CRT was significant using a two-tailed one sample *t*-test with a test value of 0. Changes in BCVA and average CRT were then compared between patients receiving Aflibercept with patients receiving Ranibizumab using a two-tailed independent samples t-test. Effect size was calculated using Cohen's *d*. Cohen defined the following interpretation as a rule of thumb: 0.2 small, 0.5 moderate, and 0.8 large [35].

Statistical analyses were made in SPSS version 23 (IBM, Armonk, NY, USA). Figures were made using Prism 7 (GraphPad Software, La Jolla, CA, USA). *P* values below 0.05 were interpreted as sign of statistical significance.

## 3. Results

### 3.1. Epidemiology: Prevalence and Presenting Characteristics

A total of 282 patients with neovascular AMD were referred to our clinic for retinal diagnosis during the year 2014. Twenty of these patients were ≥90 years, corresponding to a prevalence of 7.1% (CI 95%: 4.5 to 10.9%). Age ranged from 90 to 99 years with median 92 years and IQR 90 to 95 years. Eleven (55%) were females and nine (45%) males.

All patients presented with new neovascular AMD in one eye only, and all patients presented with a macular condition in the contralateral eye: early AMD in seven patients (35%), geographic atrophy in five patients (25%), old fibrotic AMD in five patients (25%), macular hole in one patient (5%), vitreomacular traction in one patient (5%), and subretinal drusen in one patient (5%).

Mean BCVA was 48 ETDRS letters (SD: 15 ETDRS letters). Average CRT was mean 454 *μ*m (SD: 125 *μ*m). Mean lesion size was 3563 *μ*m (SD: 1081 *μ*m) measured using the greatest linear dimension. Eleven eyes (55%) had predominantly classic lesion, seven eyes (35%) had predominantly occult lesion, and two eyes (10%) had RAP. In 15 eyes (75%), lesions were hemorrhagic in their appearance. All 20 patients were deemed eligible for treatment.

### 3.2. Treatment Adherence and Reasons for Discontinuation

For this part of the study, we included treatment results from all our patients aged ≥90 years diagnosed with treatment-naïve neovascular AMD from 2009 to 2012 (where Ranibizumab was the primary choice of treatment) and from 2014 to 2015 (where Aflibercept was the primary choice of treatment) and which during the follow-up period of 2 years was not switched from one anti-VEGF to another.

We identified a total of 116 patients treated with either Aflibercept (*n* = 54) or Ranibizumab (*n* = 62). During the 2 years follow-up, 59 patients (51%) discontinued treatment. Reasons for discontinuation are presented in Table 1 and did not differ significantly depending on choice of anti-VEGF.

**Figure 1:**
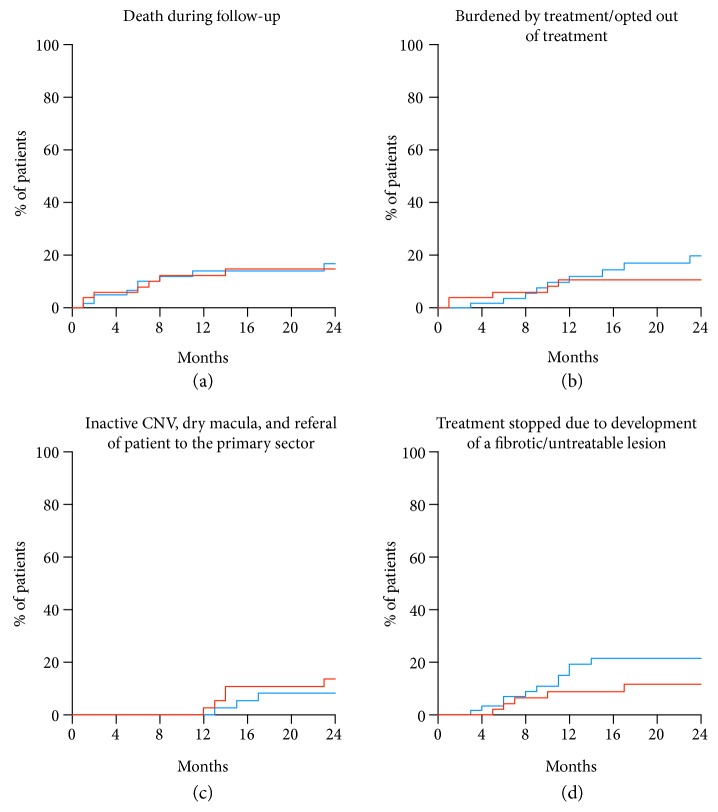
Treatment adherence in relation to different reasons for discontinuation shown in time-to-event curves. Comparing Aflibercept (red) with Ranibizumab (blue) did not show any statistically significant differences. (a) Death was an issue throughout the entire follow-up period. (b) Patients feeling burdened by the treatment opted out mostly within the first year. (c) Treatment discontinuation due to inactive CNV/dry macula was only seen after the first year. (d) Treatment discontinuation due to a fibrotic/untreatable lesion was mostly within the first year.

Using time-to-event analyses, we explored the impact of each of these factors on the treatment adherence (Figure 1). Death was an issue throughout the follow-up period. Patients who did not wish to continue treatment due to the burden of treatment made this choice within the first year. Discontinuation due to treatment results differed: discontinuation due to fibrotic/untreatable lesions was more likely to happen after the loading dose and within the first year, whereas discontinuation due to inactive CNV/dry macula happened after the first year.

### 3.3. Visual and Anatomical Outcomes

Of the 116 patients treated with either Aflibercept or Ranibizumab, 106 (91%) remained in treatment for follow-up after the loading dose phase. These patients were included for a comparison of Aflibercept and Ranibizumab efficacy in terms of visual and anatomical outcomes. Patient characteristics are summarized in Table 2 and were similar in terms of demographics, lesion characteristics, and BCVA.

Overall, anti-VEGF therapy improved the BCVA at 4 months (after the loading dose phase) (mean change 3.2 (SD: 15.5) ETDRS letters, *P* = 0.036; one sample *t*-test) and stabilized at 12 and 24 months (mean change 1.5 (SD: 16.5) ETDRS letters, *P* = 0.342; mean change −2.2 (SD: 20.1) ETDRS letters, *P* = 0.288; one sample *t*-test, resp., for 12 and 24 months). The average CRT decreased significantly and remained decreased at all follow-ups (mean change −130 (SD: 143) *μ*m, *P* < 0.001; mean change −117 (SD: 150) *μ*m, *P* < 0.001; mean change −115 (SD: 158) *μ*m, *P* < 0.001).

The mean number of treatments during 2 years was 5.7 (SD: 3.0). For Aflibercept and Ranibizumab, the mean number of treatments was 5.6 (SD: 2.9) and 5.8 (SD: 3.1), respectively. These numbers were influenced by 51% of the patients discontinuing treatment during the follow-up. Looking only at patients that continued treatment during the 2 years, the overall mean number of treatments increased to 7.2 (SD: 2.9). For Aflibercept and Ranibizumab, these numbers increased to a mean of 6.7 (SD: 3.0) and 7.9 (SD: 2.9), respectively.

Aflibercept treatment significantly improved BCVA at 4 months at mean 5.5 ETDRS letters (*P* = 0.014; one sample *t*-test) (Table 3). Although there was a small improvement at the later follow-ups at 12 and 24 months, its size was small and did not reach statistical significance (Table 3). The Δ average CRT decreased significantly at 4 months (*P* < 0.001; one sample *t*-test) and remained significantly decreased at 12 (*P* < 0.001; one sample *t*-test) and 24 months (*P* < 0.001; one sample *t*-test) (Table 3).

Ranibizumab treatment was not associated with a significant improvement or worsening of BCVA at 4 months or 12 months but lead to a significant decrease of 5.8 ETDRS letters after 24 months (*P* = 0.028; one sample *t*-test) (Table 4). The Δ average CRT decreased significantly at 4 months (*P* < 0.001; one sample *t*-test) and remained significantly decreased at 12 (*P* < 0.001; one sample *t*-test) and 24 months (*P* < 0.001; one sample *t*-test) (Table 4).

**Figure 2:**
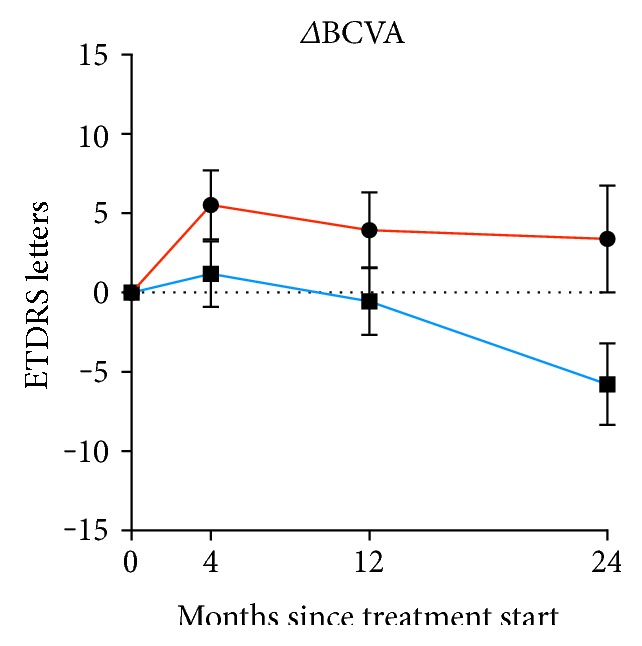
Change in best-corrected visual acuity of eyes with neovascular age-related macular degeneration in patients aged ≥90 years treated with either Aflibercept (red) or Ranibizumab (blue). Dots and whiskers indicate mean and standard error.

**Figure 3:**
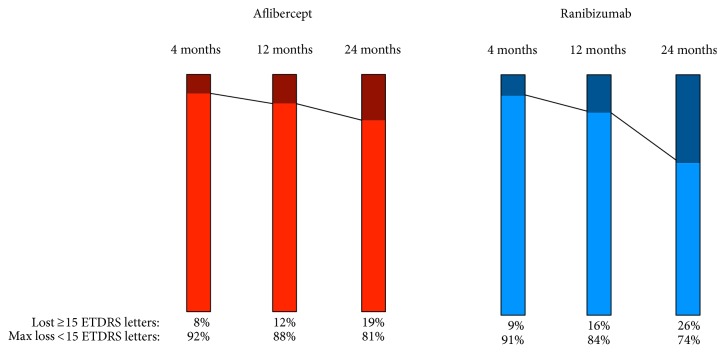
Rate of patients with neovascular age-related macular degeneration aged ≥90 years that experience a loss of ≥15 ETDRS letters in best-corrected visual acuity during treatment with either Aflibercept (red) or Ranibizumab (blue).

Overall, Aflibercept treatment was superior to Ranibizumab treatment in ΔBCVA at all time points. However, the mean differences between the groups were small initially and did not reach statistical significance until after 24 months: after 4 months (4.4 (CI 95%: −1.6 to 10.3) ETDRS letters, *P* = 0.149), after 12 months (4.5 (CI 95%: −1.9 to 10.8) ETDRS letters, *P* = 0.164), and after 24 months (9.2 (0.8 to 17.5) ETDRS letters *P* = 0.031) (Figure 2). This corresponded to Cohen's *d* values of 0.3, 0.3, and 0.5, for 4, 12, and 24 months, respectively, indicating an initially small effect size that grows to a moderate effect size at 24 months. We determined the rate of patients with loss of ≥15 ETDRS letters in each group, which also showed that the differences between the groups have a tendency of growing over time (Figure 3). After 24 months, 19% in the Aflibercept group had lost ≥15 ETDRS letters, whereas this number was 26% in the Ranibizumab group.

**Figure 4:**
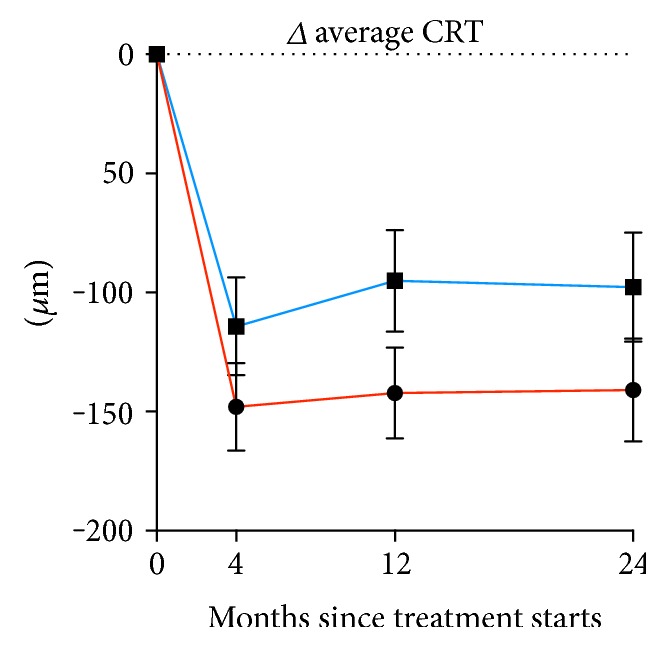
Change in average central retinal thickness of eyes with neovascular age-related macular degeneration in patients aged ≥90 years treated with either Aflibercept (red) or Ranibizumab (blue). Dots and whiskers indicate mean and standard error.

The decrease in Δ average CRT was compared between Aflibercept and Ranibizumab. Here, we saw a small but nonsignificant more decrease among those treated with Aflibercept group at all time points (34 *μ*m, 47 *μ*m, and 43 *μ*m, resp., for 4, 12, and 24, months) (Figure 4). These changes corresponded to Cohen's *d* values of 0.2, 0.3, and 0.3 for 4, 12, and 24 months, respectively, indicating a small effect size.

## 4. Discussion

Patients aged ≥90 years constitute 7% of patients with neovascular AMD—approximately one out of every 14. These patients have a high rate of treatment discontinuation, where death and burden of treatment play a considerable role. We also found that in these patients, Aflibercept therapy was superior to Ranibizumab, although the effect sizes were only small to moderate. Difference between groups in the CRT was small and not statistically significant.

Different aspects of anti-VEGF treatment may explain our results. Klettner et al. investigated the efficacy of Aflibercept, Ranibizumab, and Bevacizumab in an experimental setting using a RPE/choroid organ culture, where VEGF in the supernatant was measured throughout 7 days and using different concentration of anti-VEGFs [36]. First, Aflibercept required the lowest concentration for short-term VEGF inhibition when compared to Ranibizumab and Bevacizumab [36]. Second, one regular dose of Aflibercept inhibited VEGF completely until the 7th day, whereas VEGF could be detected after 72 hours with Ranibizumab treatment and after 12 hours with Bevacizumab treatment [36]. Brinkmann et al. compared the uptake of Aflibecept, Ranibizumab, and Bevacizumab in vitro using ARPE-19 cell cultures, where Aflibercept had significantly faster uptake when compared with Ranibizumab and Bevacizumab [37]. These findings on cellular level may not necessarily have a clinical significant impact on a broader level as seen in the VIEW studies [8]. However, considering that RPE with age becomes more fragile and develops a more potent VEGF response [23–26], patients aged ≥90 years may constitute a group where the aging process of RPE is at its utmost and where the pharmacokinetic differences between Aflibercept and Ranibizumab give rise to clinically measureable differences. However, the differences between Aflibercept and Ranibizumab on CRT had a small effect size in this study. It will be interesting to see how anti-VEGF drugs with more potent pharmacokinetic properties now in phase 3 trials, such as Abicipar/AGN-150998 (Allergan, Irvine, CA, USA) and Brolucizumab/RTH-258 (Novartis), will work on patients aged ≥90 years.

Another explanation to our findings is that Aflibercept allows eight weeks treatment intervals, which gives more flexibility from a clinical point-of-view. It is our experience that flexibility is much needed in the very old for a number of practical reasons, such as localized infections around the eye, systemic infections, fall trauma, or other conditions that may shift the priorities and the focus of the patient. For such cases, the eight weeks coverage of Aflibercept is less likely to undertreat the patients compared with the four weeks coverage of Ranibizumab. Indeed, when looking at the number of treatments, we only found that on average, ~1 additional treatment was given with Ranibizumab, underscoring our speculations of undertreatment of the Ranibizumab group.

We used a PRN regime, which some studies suggest may be inferior to the treat and extend (T&E) regimen in terms of visual outcomes [38]. In a systematic review and a network meta-analysis, Danyliv et al. compared the two regimens and found that although T&E was associated with better outcomes compared to PRN, the effect size was quite small and clinically irrelevant: ~2 more ETDRS letters at 12 months and 2-3 more ETDRS letters at 24 months [38]. This small effect size comes at a considerable cost: the T&E regimen is associated with significantly more injections [38]. However, considering that the pharmacokinetic properties of anti-VEGF drugs may play a larger role in patients aged ≥90 years, which are in risk of undertreatment, there may also be a potential for a larger gain using a T&E regime. Future studies may shed light on these aspects.

A considerable number of our patients discontinued treatment due to death or inacceptable burden of treatment. These aspects reflect the specific difficulties when dealing with patients aged ≥90 years [28]. In our study, we did not find any significant difference between the Aflibercept and Ranibizumab in the treatment discontinuation. We initially speculated that a greater number of injections might lead to a difference, but the number of injections between the groups only differed slightly. Details about burden of treatment warrant further investigation as we might be able to overcome any specific aspects proving to be an obstacle for helping our patients; for example, it would be interesting to know whether the perceived burden is due to specific factors such as the injections or the frequent visits.

Although our health system is based on a free-of-charge concept and even offers free-of-charge transportation to hospitals, actually utilizing that system may not be as straightforward in this group of very old as in others. Some of these patients informed that they did not seek help after several months, which is particularly problematic since timely treatment can be important factor for neovascular AMD [39, 40]. It is hard to speculate whether these challenges reflect a generation issue in not wanting to burden others, cognitive decline that are seen in among the very old, or a concept of accepting that vision declines with age. Interesting exploratory studies may provide better insight into the future.

Limitations should be noted when interpreting our results. This was retrospective observational study where historic cohorts were compared. Although we tried to minimize selection bias by not including participants from 2013, where Ranibizumab was used on patients with cardiovascular comorbidities; a better study design would include a randomized allocation of patients to Aflibercept and Ranibizumab. We used LOCF handle missing data due to treatment discontinuation, and in that regard, it should be noted that a considerable number of our patients discontinued treatment for different reasons. However, we do not suspect that this results in skewed data between Aflibercept and Ranibizumab since the groups did not differ in treatment discontinuation. Finally, it should be noted that the mean difference between Aflibercept and Ranibizumab was at ~2 ETDRS lines at most, and as a rule of thumb, a clinically significant difference is 3 ETDRS lines [41].

## 5. Conclusion

Patients aged ≥90 years constitute a small but important proportion of those referred for treatment of neovascular AMD. These very old patients have a high rate of treatment discontinuation, where death and burden of treatment play a considerable role. Although both Aflibercept and Ranibizumab decreased the average CRT, Aflibercept seemed superior to Ranibizumab in terms of change in BCVA after the loading dose and after 12 and 24 months in patients aged ≥90 years. Important reasons may be pharmacokinetic differences between the two drugs or the relative more flexibility of treatment every 8 weeks versus 4 weeks with Aflibercept versus Ranibizumab; however, studies with prospective and randomized design are needed for more conclusive results. We propose that the very old patients constitute a distinctive group that may warrant special attention.

## Figures and Tables

**Table 1:** Reasons for discontinuation of anti-VEGF treatment using either Aflibercept or Ranibizumab among patients with neovascular age-related macular degeneration aged ≥90 years.

	Aflibercept (*n* = 54)	Ranibizumab (*n* = 62)	*P* value
Death during follow-up, *n* (%)	7 (13)	9 (15)	0.809
Burdened by treatment/opted out of treatment, *n* (%)	6 (11)	10 (16)	0.434
Inactive CNV, dry macula, and referral of patient to the primary sector, *n* (%)	5 (9)	3 (5)	0.470
Treatment stopped due to development of a fibrotic/untreatable lesion, *n* (%)	6 (11)	11 (18)	0.314

*P* values were calculated using the *χ*^2^-test for all categories, but *inactive CNV, dry macula, and referral of patient to the primary sector* was calculated using Fisher's exact test due to groups with <5.

**Table 2:** Baseline factors of patients with neovascular age-related macular degeneration aged ≥90 years enrolled in Aflibercept or Ranibizumab treatment, which remained in treatment for at least the follow-up after the loading dose phase.

	Aflibercept (*n* = 49)	Ranibizumab (*n* = 57)	*P* value
Age, years, median (IQR)	91 (90 to 93)	91 (90 to 92)	0.400
Gender, *n* (%)			0.370
Female	35 (71)	45 (79)	
Male	14 (29)	12 (21)	
BCVA, ETDRS letters, mean (SD)	50 (14)	48 (18)	0.510
Average CRT, mean (SD)	433 (130)	445 (114)	0.660
Lesion type, *n* (%)^†^			0.309
Predominantly classic	16 (35)	18 (34)	
Predominantly occult	25 (54)	34 (64)	
Retinal angiomatous proliferation	5 (11)	1 (2)	

IQR: interquartile range; SD: standard deviation; BCVA: best-corrected visual acuity; CRT: central retinal thickness.^†^No data on lesion type for three patients in the Aflibercept group and four patients in the Ranibizumab group due to allergies to the contrast agents, lack of cooperation, or inaccessible data. *P* values were calculated using the Mann–Whitney *U* test for age, *χ*^2^-test for gender, independent samples *t*-test for BCVA and average CRT, and Fisher's exact test for lesion type due to groups with <5.

**Table 3:** Two-year results on Aflibercept treatment for neovascular age-related macular degeneration in patients aged ≥90 years.

	Aflibercept (*n* = 49)	
	Mean (95% CI)	*P* value
ΔBCVA, ETDRS letters		
4 months	5.5 (1.1 to 9.9)	0.014
12 months	3.9 (−0.9 to 8.8)	0.106
24 months	3.4 (−3.4 to 10.2)	0.320
Δ average CRT, *μ*m		
4 months	−148 (−185 to −111)	<0.001
12 months	−142 (−181 to −104)	<0.001
24 months	−141 (−185 to −97)	<0.001

BCVA: best-corrected visual acuity; CRT: central retinal thickness; CI: confidence interval. *P* values were calculated using the one sample t-test with test value = 0.

**Table 4:** Two-year results on Ranibizumab treatment for neovascular age-related macular degeneration in patients aged ≥90 years.

	Ranibizumab (*n* = 57)	
	Mean (95% CI)	*P* value
ΔBCVA, ETDRS letters		
4 months	1.2 (−3.0 to 5.3)	0.570
12 months	−0.5 (−4.8 to 3.7)	0.800
24 months	−5.8 (−10.9 to −0.6)	0.028
Δ average CRT, *μ*m		
4 months	−114 (−155 to −73)	<0.001
12 months	−95 (−138 to −52)	<0.001
24 months	−98 (−143 to −52)	<0.001

BCVA: best-corrected visual acuity; CRT: central retinal thickness; CI: confidence interval. *P* values were calculated using the one sample *t*-test with test value = 0.
